# Mass Spectrometric Immunoassays in Characterization of Clinically Significant Proteoforms

**DOI:** 10.3390/proteomes4010013

**Published:** 2016-03-17

**Authors:** Olgica Trenchevska, Randall W. Nelson, Dobrin Nedelkov

**Affiliations:** The Biodesign Institute, Arizona State University, Tempe, AZ 85287, USA; randal.nelson@asu.edu (R.W.N.); dobrin.nedelkov@asu.edu (D.N.)

**Keywords:** biomarkers, immunoaffinity, mass spectrometry, posttranslational modifications, top-down analysis

## Abstract

Proteins can exist as multiple proteoforms *in vivo*, as a result of alternative splicing and single-nucleotide polymorphisms (SNPs), as well as posttranslational processing. To address their clinical significance in a context of diagnostic information, proteoforms require a more in-depth analysis. Mass spectrometric immunoassays (MSIA) have been devised for studying structural diversity in human proteins. MSIA enables protein profiling in a simple and high-throughput manner, by combining the selectivity of targeted immunoassays, with the specificity of mass spectrometric detection. MSIA has been used for qualitative and quantitative analysis of single and multiple proteoforms, distinguishing between normal fluctuations and changes related to clinical conditions. This mini review offers an overview of the development and application of mass spectrometric immunoassays for clinical and population proteomics studies. Provided are examples of some recent developments, and also discussed are the trends and challenges in mass spectrometry-based immunoassays for the next-phase of clinical applications.

## 1. Introduction

Protein biomarkers are utilized in clinical diagnosis, prognosis and treatment monitoring of many diseases [1]. Today, numerous protein lab tests are being used to provide clinically relevant information for evaluation of physiological states and existence of pathological condition. In biological specimens that are commonly used for biomarker assessment (such as human serum, plasma and urine), proteins are present in a large span of concentrations [2]. Therefore, detection and analysis of protein biomarkers is very complex and challenging.

The majority of methods for analysis of protein biomarkers in clinics are designed for intact protein analysis and provide information about the abundance of the targeted protein in the biological sample [3,4]. These methods primarily use immunoaffinity capture of the protein, followed by detection. Depending on the type of detection, several methods, such as enzyme-linked immunosorbant assays (ELISAs), radioimmunoassays (RIAs), electrochemiluminescence immunoassays, and immunoturbidimetry/immunonephelometry assays [5], have been adopted in clinical laboratories for protein biomarker analyses.

Many proteins are known to exist as multiple variants (or proteoforms) *in vivo* [6,7]. The term “proteoform” has recently been adopted to explain protein derivatives which originate from posttranslational processing, genetic polymorphisms and mutations, or truncations [8]. Posttranslational modifications (PTMs) are chemical alterations in protein structure, typically catalyzed by substrate specific enzymes, which are under strict control. Combinations of different, sub-stoichiometric PTMs introduce the heterogeneity of the protein population. Currently, there are more than 300 known types of PTMs [9]. The frequency and abundance of different proteoforms are influenced by genetic predisposition, as well as environmental factors. Since the proteome is a dynamic system and changes continuously, proteoforms distribution can be viewed as a fingerprint of the physiological state at a specific time. Studying PTMs is necessary to better understand the proteome dynamic changes and the effects different PTMs have on cellular phenotypes. Proteins that undergo posttranslational processing exhibit changes in their molecular weight. Some PTMs add to the protein mass (e.g., phosphorylation + 80 Da, acetylation + 42 Da, cysteinylation + 119 Da, glycation + 162 Da, different glycosylation patterns add from +160 to more than 1 kDa). Truncations, on the other hand, cause shifts towards lower molecular weights, as a result of the removal of a single *N*- or *C*-terminal amino acid, or a small side chain in the amino acid sequence of the protein.

Posttranslational modifications can be tracked as disease markers or used as molecular targets for developing target-specific therapies. They have a profound effect on the stability, activity, and pharmacokinetics of many therapeutic proteins and are used in clinical diagnosis [10]. For example, hemoglobin A1C (HbA1C) is a known proteoform that is used on regular basis for monitoring glucose clearance in type 2 diabetes patients [11]. Approximately 10% of the Food and Drug Administration (FDA) approved tests for clinical protein biomarkers in plasma or serum are designed for analysis of PTMs [12].

Numerous methods used in proteomics, from gel electrophoresis and affinity-based analytical methods to structural interaction analyses and protein crystallography, can be applicable for analysis of PTMs [13]. The main drawback for these methodologies is the complicated sample preparation and processing, lack of high-throughput, and inability to unambiguously detect novel proteoforms. Mass spectrometry (MS)-based protein assays hold great potential for *in vitro* detection of protein biomarkers. MS-based methods measure the molecular mass of the protein target, which is a unique property of each protein. The positive aspect of MS is that it is the only current detection method that can unambiguously provide information about specific protein structural modifications, without a priori knowledge of the modification. The protein mass of each fully expressed and functional protein contains information about the gene that encodes the protein and the post-expression processing that the protein undergoes. Hence, changes in the gene sequence and/or post-expression processing are reflected in the mass of the whole protein, and can be detected via MS.

There are two main strategies to analyzing proteins using MS. In the bottom-up approach, proteins present in biological samples are digested using proteolytic enzymes and their constituent peptide fragments are detected via MS and more often tandem MS (MS/MS) [14,15,16]. Methods, such as stable isotope standards and capture by anti-peptide antibodies (SISCAPA) [17,18] and stable isotope labels with amino acids in cell culture (SILAC) [19] are geared towards detection of proteolytic peptides as surrogate measures for protein quantification. The protein identification and/or quantification are oftentimes based on the positive identification of selected peptides, leaving a large part of the protein sequence un-assessed. A consequence of the limited sequence coverage in these bottom-up proteomics approaches is possible loss of information about PTMs—(proteoforms without *a priori* knowledge of their existence are not detected and analyzed). This major drawback has initialized development of modified and improved bottom up strategies that are able to assess PTMs by implementing sample pretreatment and/or specific peptide targeting [20]. Different labels have been used to mark the peptide of interest, such as heavy isotope labels (in SILAC) [21], chemical labels (in isotope-coded protein labeling—ICPL) [22], dimethyl labels, tandem mass tags (TMT) [23], and isobaric labels (in isobaric tags for relative and absolute quantification—iTRAQ) [24]. Some of these labels are introduced in the sample prior to digestion, while others are used to directly label the peptides following enzymatic digestion. Due to the low abundance of PTMs in comparison to the originating proteins, multiple approaches implement peptide enrichment to decrease the sample complexity and also to assist in detection of the low abundance peptides [25,26]. Currently, more in-depth analysis of proteoforms, as well as identification of novel PTMs is done by utilizing tandem mass spectrometry (MS/MS) methods [27,28]. Tandem mass spectra are obtained by fragmentation of a precursor peptide ion (chosen form a mass spectra obtained after enzymatic digestion on the protein target) into daughter ions, utilizing either collision-induced dissociation (CID) or electron capture dissociation (ECD) [29,30,31,32,33]. Tandem MS can provide two types of information; it can confirm the protein identification based on the daughter ions and characteristics of the obtained peptide map and primary structure [34]. In addition, the obtained information from the MS/MS allows for exact localization of posttranslational or other modification sites, thus distinguishing between proteoforms that have the same mass shifts [28,35]. With the advanced data analysis programs, MS/MS can be used to confirm the PTM identification and minimize the false positive proteoform identification [36]. In spite of the multiple processing and analysis steps associated with tandem MS methods, they have been used in numerous studies for PTM mapping and identification [37,38,39,40].

Top-down MS-based approaches analyze intact proteins, without previous fragmentation to peptides [41,42]. These methods are better suited for proteoforms detection because they detect the mass of the intact proteoforms, and cover the putative modifications in the entire protein sequence. These methodologies can be suited to identify PTMs, gene variants as well as transcript variations and the relative occupancy of the modification sites. The major advantage of the top-down approaches is the simple sample preparation—when coupled with immunoaffinity capture of the protein target, the specific MS detection enables for identification of all the present proteoforms without complex sample pretreatment. Following isolation or extraction, intact proteins are ionized (either by electrospray ionization—ESI, or matrix-assisted laser desorption ionization—MALDI) or fragmented (in collision-induced dissociation—CID or infrared multiphoton dissociation—IRMPD), and the resulting ions are analyzed using quadrupole time-of-flight (QTOF), fourier transform ion cyclotron resonance (FT-ICR), TOF/TOF or orbitrap MS detectors [43].

**Figure 1:**
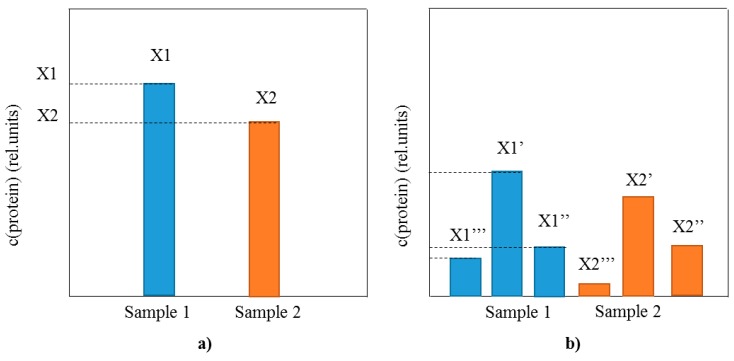
Schematic representation of the differences between single protein biomarker analysis using (**a**) conventional immunoassays (total protein concentration is measured); and (**b**) top-down MS-based immunoassays (protein profile and all proteoform concentrations are measured).

Implementation of MS as a detection method for intact protein biomarkers analyses provides with the much needed information about the profile of posttranslationally modified proteins that is lacking using conventional immunoassays (Figure 1). As opposed to the sole comparison of the total protein concentration between samples (as in conventional immunoassays) (Figure 1a), MS-based methods have the ability to perform extensive analysis of the protein profile, including the distribution of existing proteoforms (Figure 1b). The proteoform distribution represents a fingerprint of the present physiological state related to the protein target, as well as an insight into the intrinsic protein characteristics. In a single analysis MS enables for quantification of the protein of interest, but also determines the presence and concentration of additional proteoforms. This added benefit of the top-down MS to perform both qualitative profiling and quantitative analysis of multiple proteoforms is a step forward in the protein biomarker assaying.

Today, several top-down MS-based technologies are used for identification and quantification of proteoforms in clinical diagnostic laboratories. Analysis of carbohydrate deficient transferrin (CDT) is performed for chronic alcohol abuse and, in combination with apolipoprotein C-III (apoC-III) proteoforms, for congenital disorders of glucosylation (CDG) [44]. These assays utilize immunoaffinity capture of transferrin (in CDT) and transferrin and apolipoprotein C-III (in CDG) with antibodies, followed by electron spray ionization mass spectrometric (ESI-MS) detection. Results are presented as different ratios between the glycosylated transferrin and apoC-III. Single reaction monitoring (SRM) LC-MS methods have been developed for clinical analysis of intact insulin-like growth factors 1 (IGF1) and 2 (IGF2) [45,46], and insulin [37] and their proteoforms. These assays show the benefit of top-down MS-based approaches and are great examples of their potential and applicability for clinical assaying.

In the past 20 years, our group has been exploring the potential of proteoforms analyses for different protein targets using immunoaffinity MS-based methodologies. As a result, a top-down approach named mass spectrometric immunoassay (MSIA) has been developed and utilized in analysis of numerous proteins and proteoforms.

## 2. Mass Spectrometric Immunoassay Principle

**Figure 2:**
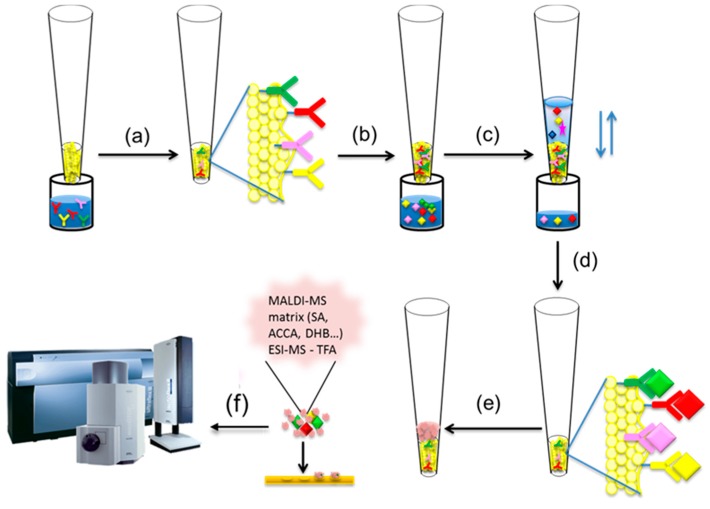
Mass spectrometric immunoassay (MSIA) workflow (**a**) affinity pipettes derivatization with antibody(ies); (**b**) introducing affinity pipette in biological sample; (**c**) protein(s) extraction; (**d**) rinsing non-specifically bounded substances from the affinity pipette; (**e**) protein(s) elution with matrix; (**f**) protein detection using MALDI-TOF MS or ESI MS

Mass spectrometric immunoassay (MSIA) was first conceptualized by Nelson *et al.* [47,48]. It is a top-down approach for intact protein analysis where a micro-scale immunoaffinity capture is combined with either ESI or MALDI-TOF mass spectrometric detection (Figure 2). Single or multiple antibodies towards the targeted protein(s) are surface-immobilized in small, porous columns that are fitted at the entrance of a pipettor tip, forming an affinity pipette (Figure 2a).

By sample aspiration and dispense through the tip, a close contact between the immobilized antibody and the protein present in the biological sample is obtained (Figure 2b). This cycle of aspirations/dispenses is repeated until enough protein is bound to the antibody (Figure 2c). Once the protein is captured, the affinity pipette is washed to remove any loosely associated sample components and non-specifically bounded proteins (Figure 2d). A small volume of MALDI matrix is then aspirated into the affinity pipette (Figure 2e) and used to quickly deposit the protein-containing eluate directly onto a MALDI target for subsequent MALDI TOF MS analysis (Figure 2f). If the sample is analyzed using ESI MS, than the protein elution is done with 0.4% TFA.

The dual specificity character of the mass spectrometric immunoassays offers a unique advantage over conventional enzymatic immunoassays. The primary antibody utilized for the protein affinity capture provides the first level of specificity. In addition, while sandwich ELISAs rely on binding of a secondary antibody for protein detection, MSIA offers direct readout of the protein molecular mass, which is an intrinsic characteristic of each protein. A signal in the mass spectrum at an *m*/*z* value that corresponds to the protein theoretical molecular mass is a clear indication of the successful protein extraction from the sample.

MSIA has potential to overcome several of the drawbacks associated with MS-based protein assays—the simple sample preparation, the specific detection and high throughput. Because of the “gentle” sample pretreatment (no digestion, or use of potent solvents and harsh chemicals), MSIA analyzes of intact proteins hold a great potential for preserving the protein amino acid sequence for further exploring its intrinsic properties. The detection and identification of the protein is based solely on the molecular mass, which adds the necessary specificity. Additionally, MSIA can be executed on an automated robotic platform, which makes it possible for parallel analyses of multiple samples. Provided below are details about the method development and validation protocol used in MSIA, which will further outline its potential for analysis of clinically significant proteins.

### 2.1. Method Development with Mass Spectrometric Immunoassay

**Figure 3:**
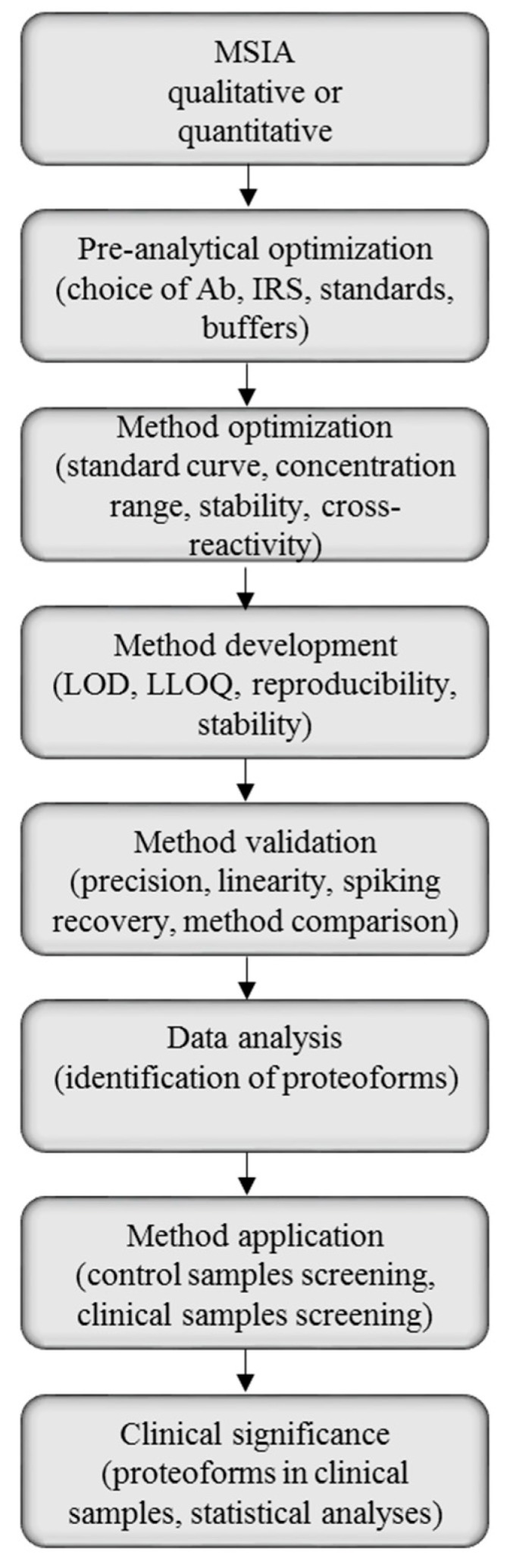
Mass spectrometric immunoassay (MSIA) method development, validation and application workflow.

Mass spectrometric immunoassay can be suited for both qualitative and quantitative analysis of single and multiple proteins, thus, the method development will be highly dependent of the purpose of the analysis. There are, however, general steps in the MSIA protocol, and those can be used as guidelines for method development and validation (Figure 3).

In the pre-analytical optimization when developing a qualitative MSIA, the initial step is the choice of antibody(ies) towards the targeted protein (for a single protein assay) or proteins (for multiplexed assays). Depending on the availability, this step usually requires testing of several commercially available antibodies, or use of custom-made antibodies. For known proteins that are tested regularly in clinics using immunoassays, the primary antibody from ELISA can be used for MSIA. For proteins that are known to have posttranslational modifications, polyclonal antibodies should be used, due to the broad specificity. Each antibody molecule has two binding sites, but the polyclonal antibody contains multiple antibodies—each potentially targeting different epitope of the protein. Monoclonal antibodies, on the other hand, are more selective, and evaluation of their efficiency needs to be proven prior to their utilization in MSIA. Selected antibodies are then used to derivatize the immunoaffinity pipettes. The amount of antibody in each pipette depends on the properties of the antibody and the targeted protein, its binding kinetics, as well as the estimated protein abundance in the biological specimen. The quantity of antibody needs to be sufficient to capture the targeted protein from the analyzed sample. This amount can be optimized experimentally. The selection and optimization of the antibody is one of the crucial steps for developing a successful MSIA.

The next step in the MSIA method development includes analyzing the antibody selectivity. The antibody of choice needs to be able to capture only the targeted protein from the sample, but not other biomolecules. To ensure the least interferences, multiple antibodies for the protein target should be compared for their selectivity of protein capture. To analyze the affinity towards the targeted protein, antibodies need to be tested with different dilutions of protein target standard (synthetized with high purity or purchased). If available, protein depleted plasma (plasma without the targeted protein) may be used to check for cross-reactivity of the antibody.

The derivatized affinity pipettes are next introduced to the biological sample. This step differs significantly depending on the type of analysis. In qualitative MSIA, optimization of the sample preparation is performed. For quantitative MSIA, there are several pre-preparation steps before subjecting to the sample preparation (method optimization phase, Figure 3). This includes choosing a protein standard, as well as an internal reference standard (IRS) for quantification. In quantitative MSIA the choice on an IRS is one of the crucial steps in the method development. A single protein may serve as an IRS if an antibody toward that protein is co-immobilized with the antibody toward the targeted protein, and the analytical samples are spiked with constant amounts of that IRS. An important prerequisite for the IRS is that it cannot be endogenous to human plasma or serum—its spiked concentration in the analytical samples should always be constant and not influenced by the human serum components. The IRS should also produce signals in the mass spectra that are in close proximity of the targeted protein signal, so that the same MS acquisition parameters can be used for both proteins. Another approach is to use homologous proteins from other animal species (such as equine protein) [49], or protein derivatives (His-tag modified proteins, or methionine (Met) modified proteins) [50,51] as IRS. These homologs are recognized by the anti-human protein antibody and are spiked into the analytical sample. The single antibody then retrieves both, the target protein and the IRS, and they register in the same region of the mass spectra, but at a slightly different *m*/*z* value.

When using exogenous IRS, the general rule for affinity pipettes derivatization is that they should have higher affinity for the targeted protein than the IRS because the targeted protein varies in concentration across the samples, while the IRS can be spiked and kept constant at levels sufficient to saturate the anti-IRS antibody and produce constant signal in the mass spectra.

Protein standards (native and/or recombinant) can be utilized for generation of standard curve for quantification. Standard curves are generated with serial dilution of a protein standard, in a concentration range that is experimentally optimized. Linear standard curve should be fitted in a wide or narrow concentration range, depending on the possible fluctuations of the targeted protein in the biological specimen. The range of the standard curve should be sufficient to analyze the protein from biological samples prepared in corresponding dilution. A common approach is to optimize the concentration of the IRS and the protein standards in parallel, using empirical iterations. In a two-step approach, initially the concentration of the protein standard is kept constant, while the IRS concentration is varied, until the anti-IRS antibody is completely saturated (resulting in constant signal in the mass spectra). Next, keeping the IRS concentration constant, the protein standard concentration is varied, and a standard curve range is established.

The biological specimen is prepared depending on the estimated physiological concentration of the targeted protein. For high abundance proteins, samples require higher order dilutions (100×, 1000×) and lower initial sample volume, whereas low abundance protein targets require higher volumes of undiluted sample. The sample preparation before the affinity capture usually requires only simple dilution in sample buffer, just prior to analysis and does not involve digestion and/or extensive sample preparation.

MSIA is suited to analyze sample volumes as little as few microliters (μL) to up to a few milliliters (mL). The affinity pipettes are introduced in the samples, and affinity capture of the targeted protein is achieved by keeping the antibodies on the pipettes in continuous contact with the proteins in the biological sample. This is accomplished by repeated cycles of aspiration and dispense of a small sample volume through the pipettes (typically, 50–100 μL). The continuous flow of sample through the pipette enables for direct contact and capture of the targeted protein to the antibody. The cycle counts can easily be adjusted on an automated platform enabling for sufficient interaction between the protein and the antibody for prolonged times.

All analyses can be executed manually on a single-channel or 8- and 12-channels pipettors, or on a 96-channel automatic pipettor, for additional high-throughput capacity. The samples, solvents and buffers, as well as the MALDI matrix, are placed on the automated pipettor platform, and with a computer-controlled program, all MSIA steps can be executed automatically. The entire process from derivatized affinity pipettes, to mass spectra analysis takes approximately 1 hour for 96 samples in parallel, making the full capacity of around 1000 samples per day [52].

Depending on the mass detection method, captured proteins are eluted from the bounded antibody in the affinity pipette with MALDI matrix (for MALDI TOF MS) or trifluoroacetic acid—TFA (for ESI MS). In the case of MALDI-TOF MS, the MALDI matrix (state what is the chemical compound most commonly used) is aspirated several times through the pipette. The acidic base of the matrix solvent disrupts the antibody-protein bond, releasing the protein from the affinity pipette to the target plate, in preparation for the MS analysis.

Mass spectra are next acquired using either MALDI TOF or ESI MS. The choice and optimization of MS detection method depends on several factors: (1) the characteristics of the protein target (MW, known modifications, *etc.*); (2) type of analysis (qualitative, quantitative); and (3) availability. Usually MALDI TOF (or TOF/TOF) MS is used for analysis of peptides and proteins with MW < 30 kDa. ESI QTOF MS has been applied to high molecular weight proteins as well as peptides and/or proteins that are known to be carboxylated (contain carboxylation sites in their sequence) [53,54]. In some cases, both MALDI and ESI MS may be used as complement ionization methods for addressing protein heterogeneity [53,55]. Following mass spectra acquisition (in MALDI) and after deconvolution (in ESI), signals from the targeted protein from each biological sample are evaluated. The signal from the targeted protein must be present at the exact *m*/*z* value as the theoretically expected, and with signal-to-noise (S/N) > 5 when using corresponding antibody.

In the method development phase, the antibody selectivity is examined by analyzing the binding between the antibody and the protein form the samples. The optimized MSIA is expected to result in signal(s) in the mass spectra originating from the targeted protein that appear when the sample is analyzed using the derivatized affinity pipettes with the corresponding anti-target antibody, but must not be present when no antibody is present, or when affinity pipettes derivatized with other antibodies are used. In addition, the background dependent of the assay execution must be maintained to <50%.

To account for possible physiological differences in the specimen, prior to application onto clinical samples, the targeted MSIA can be evaluated in fresh, pooled, hemolyzed and lipemic plasma samples. Also, the effect of anticoagulants can be examined by analyzing Na-EDTA, K-EDTA, Na-citrate, and heparin plasma, as well as serum samples. Freeze and thaw stability are examined by analyzing the protein in single sample after multiple freeze-thaw cycles. Bench-top stability is further used to evaluate the stability of the protein target during the sample preparation and assaying.

Following method optimization, MSIA is applied in screening of the targeted protein(s) in healthy population, as well as in clinical cohorts. By performing cross-sectional and/or longitudinal screenings, potential protein biomarkers can be assessed in both physiological and pathological context.

### 2.2. Method Validation for Quantitative Mass Spectrometric Immunoassay

Assay validation is a crucial step to verify the consistency, reproducibility, and high-throughput of the developed MSIA, as well as to confirm the feasibility for further application in analysis of clinically significant targets. There are numerous protocols that focus on addressing the benefits and standardizing the criteria for application of MS-based methodologies for clinical assays [56,57,58]. Carr *et al.* present a detailed review of a validation platform according to the established “fit-for-purpose” approach [59]. Initially, the reproducibility of the response of the calibration curves for each of the proteins needs to be addressed by inter- and intra-day precision experiments, performed by analyzing a minimum of 20 human plasma samples in relevant matrices (e.g., biological replicates, and pooled samples). All experiments are performed in a minimum of triplicate measurement (up to five replicates in a single run) to address reproducibility. The analytical validation also includes measurement of assay precision, definition of the linear range and determination of the limit of detection (LOD) and lower limit of quantification (LLOQ) using standardized protocols [60]. Linearity experiments are done by serially diluting samples with known protein concentration, analyzing them with MSIA, and comparing the results with those expected. Spiking recovery experiments are further performed by spiking the protein target standard into biological sample and retrieving the expected standard concentration with the sample protein in a single run. Coefficients of variation (CV) levels of <10%, and linearity and recovery between 85% and 115% are accepted when comparing the obtained measurements with the theoretically expected in the MSIA platform.

As a final step of the method validation, each developed MSIA is benchmarked against commercially available ELISA (or other immunoassay) for the protein target. Method comparison is done by analyzing a minimum of 20 plasma samples (in duplicates) with MSIA and commercially available ELISA in parallel. Obtained results are then compared using Bland-Altman [61] and Passing-Bablook [62] method comparison tests.

### 2.3. Challenges and Limitations of Mass Spectrometric Immunoassay

There are several limitations that make method development in MSIA challenging. Majority of concerns are associated with all MS-based methodologies, since most top-down and some bottom-up approaches utilize either MALDI or ESI MS. Optimizations are required regarding the ionization efficiency, reproducibility and high-molecular weight (HMW) protein detection using MALDI and ESI MS. In practice, the ionization is highly dependent on the protein target and its composition. For example, MALDI is more suitable for protein targets with more basic and aromatic amino acids in the sequence, whereas ESI can more efficiently be applied for ionization of hydrophobic proteins [26,63,64]. Regarding the molecular weight, MALDI is more suitable for small and medium size proteins (up to 30 kDa), but does not have the required resolution that ESI has for HMW proteins. For quantitative measures, the major concerns are regarding the reproducibility of the analyses [65]. Controls in every step of the method development need to be used. Additionally, the addition of the IRS is beneficial to account for the between and within run variability.

As an immunoaffinity capture-based methodology, the primary limitation of MSIA, is the availability of high quality antibodies. The selected antibodies must be with high purity; for example, polyclonal antibodies need to be subjected to immunogen-based affinity purification and, monoclonal antibodies should be affinity purified at least by protein A or protein G-based affinity purification. The low performance of the antibody is addressed early in the method development and can be overcome by testing other sources, or use mixture of antibodies towards the same target. Additionally, formulation buffers must not contain functional groups that might affect the covalent binding of antibodies to the immobilization surface so, for example, TRIS or carrier proteins cannot be used when coupling antibodies to carboxyl-functionalized tips using carbonyldiimidazole during the derivatization [66].

Other limitations associated with the experimental design and the sample preparation, such as storage, absence of signals in the mass spectra, poor quality metrics may be addressed by carefully choosing the analytes (buffers, standards, and samples) and controlling every step during the method development.

## 3. Identification and Analysis of Proteoforms with Mass Spectrometric Immunoassay

Mass spectrometric immunoassay has a great advantage over the conventional immunoassays because of the way it looks at proteins. The soft MALDI ionization preserves the protein in its native form, and produces signals in the mass spectra originating from the full-length form of the targeted protein, as well as different proteoforms in a present state. MSIA has the potential to identify known, as well as novel PTMs by analyzing the signals that appear in the mass spectra. The PTM signal can be initially assigned by accurate measurement of the mass shift and knowledge of the protein sequence. After identification, further quantification of the proteoforms is enabled with the use of the internal reference standard and standard curves, and is calculated as a percentage of the total protein concentration. Mapping the detected and quantified novel proteoforms aids the general knowledge about the analyzed proteins.

This approach is useful because a clinical condition, or an exogenous factor, such as introducing some medication, may trigger changes in the metabolic processes in the cells, inducing more or less specific enzymes secretion. The enzymes may induce cleavages in protein targets, resulting in changes in the distribution and abundance of different proteoforms.

Some of the widely utilized biomarkers of disease, such as cystatin C [67,68], prostate-specific antigen [69] or cardiac troponin I [70,71], are known to exist as several forms *in vivo*. Other proteins, such as hemoglobin, serum amyloid A and numerous membrane proteins and enzymes, have genetic polymorphisms that induce expression of variable proteoforms, each unique for an individual. MSIA has the ability to identify and further quantify such heterogeneity, which is presented further through the following example.

Serum amyloid A protein (SAA) is coded by 4 genes (*SAA1, SAA2, SAA3* and *SAA4*), 3 of which are expressed in humans (*SAA1, SAA2* and *SAA4*), and 2 of which direct synthesis of 5 (SAA 1.1, SAA 1.2, SAA 1.3, SAA 1.4 and SAA 1.5) and 2 (SAA 2.1 and SAA 2.2) SAA proteins [72,73]. Moreover, truncated proteoforms of SAA have been known [74,75,76].

**Figure 4:**
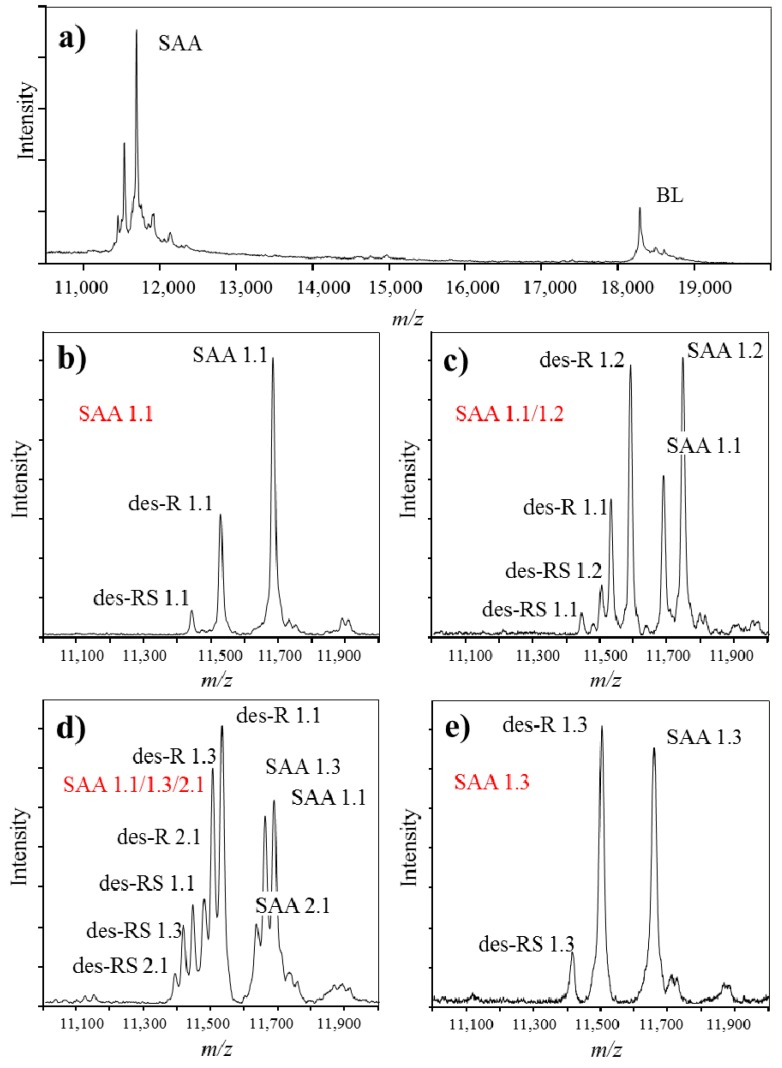
Example mass spectra obtained using MSIA, from different individuals expressing single and multiple SAA polymorphic variants. (**a**). MALDI-TOF mass spectra obtained from analysis of SAA in human plasma sample, using beta lactoglobulin (BL) as an internal reference standard; (**b**)–(**e**). Close-up of SAA from 4 different human plasma samples; (**b**) SAA 1.1 polymorphic variant (expressed are signals from the native SAA 1.1, as well as two SAA 1.1 proteoforms lacking one (des-R) and two (des-RS) N-terminal amino acids); (**c**) SAA 1.1/1.2 polymorphic variant (two SAA polymorphic variants are expressed, together with the corresponding truncated proteoforms); (**d**) SAA 1.1/1.3/2.1 polymorphic variant (three SAA polymorphic variants are expressed) and (**e**) SAA 1.3 polymorphic variant. Note that beside the originating full-length SAA protein, all samples present with truncated proteoforms; SAA proteoforms in the figure are labeled according to the revised nomenclature for serum amyloid A by the nomenclature committee of the international society of amyloidosis [84].

Immunoassays that are commonly used in clinics for assessment of SAA protein, provide with quantitative readout that equals the total SAA concentration. However, they do not possess the capability to distinguish between different SAA polymorphic variants and truncated proteoforms in a single analysis. MSIA can be successfully used in such cases. SAA is a crucial inflammation biomarker related to numerous clinical conditions [73,77,78,79,80]. In addition, expression of certain SAA polymorphic variants has been associated with differences in the basal SAA concentration [81,82]. Therefore, it is justified to develop an assay for analysis of SAA proteoforms. In our previous work, we have developed MSIA for analysis of SAA in human plasma samples (Figure 4) [83]. Presented in Figure 4a is a typical mass spectra obtained with MSIA for SAA. Present in the mass spectrum are: BL signal at *m*/*z* = 18,281 Da, originating from beta lactoblobulin (an exogenous protein standard that was used as an IRS), as well as multiple signals in the *m*/*z* range between 11.2 and 11.8 kDa, representing various proteoforms of SAA. Shown in Figure 4b–e are MALDI-TOF mass spectra in a narrower *m*/*z* range, showing SAA from four different human plasma samples. Note that multiple signals originating from *SAA1* and *SAA2* gene products are present. The sample in Figure 4b contains only the SAA 1.1 proteoform, a product of *SAA1* gene, whereas Figure 4e contains a single SAA 1.3 protein. Samples in Figure 4c,d contain multiple SAA polymorphic variants as shown.

In addition to the genetic variations that were found to be different among the samples, SAA presented with additional proteoforms. Using MSIA, truncated SAA proteoforms, lacking one, (des-R SAA), two (des-RS SAA) or more amino acids from the *N*-terminal of the native protein sequence were identified and analyzed (Supplementary Table S1). Truncated proteoforms were confirmed for all identified SAA proteins, regardless of the polymorphic variant, as presented in the mass spectra in Figure 4.

**Figure 5:**
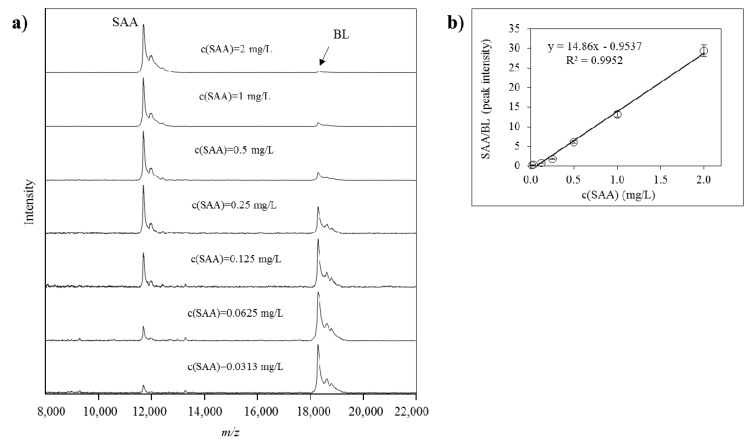
(**a**) Mass spectra from serum amyloid A (SAA) standard in different dilutions, and beta lactoglobulin (BL) as an internal reference standard (IRS), obtained using MSIA; (**b**) generated standard curve from SAA and BL as an IRS.

Quantification of all identified SAA proteoforms (native and truncated) was performed using a generated standard curve. Each standard curve was constructed from the ratio between the intensities of the SAA standard signal and the BL signal (SAAstd/BL, y-axis) against the concentration of the SAA standard (c(SAA)std, x-axis). Linear fitted curve was constructed and the generated equation was used to calculate the total SAA concentration in the plasma samples. The concentration of each separate SAA proteoform was calculated as a percentage from the total SAA, as calculated from the signal intensity of each PTM peak *vs.* the sum of all peak intensities in the sample. An example of a SAA standard curve together with the corresponding mass spectra from the standards is presented in Figure 5.

Additionally, for calculation of the concentration of SAA proteoforms in the samples, every generated standard curve (with each run) is used to address the within-assay variability. A control sample is analyzed in triplicates with each sample set and the same standard set, and the CV is calculated from the mean and standard deviation between the retrieved SAA concentrations. Coefficient of variation lower than 10% must be obtained in order to accept the results from the samples.

## 4. Mass Spectrometry Immunoassay for Clinically Significant Proteoforms

Since 1995, our group has published extensively on MSIA including core technology conception and development [47], assay development for dozens of human proteins [85,86,87,88], novel data modeling approaches [89], and also demonstrated ultra-high throughput application for >1000 samples per day intended for clinical use [52]. Listed in Table 1 are mass spectrometric immunoassays for human proteins expressing various proteoforms.

MSIA qualitative assays have identified novel proteoforms originating from several human plasma proteins, annotated entries in the Uniprot database for apolipoprotein A-I (P02647), apolipoprotein A-II (P02652), C-reactive protein (P02741), insulin-like growth factor II (P01344), retinol binding protein (P02753), serum amyloid A (P02735), and serum amyloid P (P02743).

Protein heterogeneity and distribution has been analyzed in wide-range proteoforms-discovery studies, in order to explore the high throughput potential of MSIA [115,116]. The range of proteoforms concentrations in large cohorts (more than 500 samples) was analyzed in healthy population [112], in an endeavor termed population proteomics [117,118]. Population proteomics studies have provided information about the distribution and frequency of abundance of proteoforms originating from proteins such as cystatin C, transthyretin, beta 2-microglobulin and retinol binding protein [114,115]. In addition, changes in protein profiles in time have been monitored, by performing a longitudinal study of the proteoform distribution [112].

More recently, our research has been focused on clinical biomarker discovery/rediscovery. MSIA has been applied to few well-characterized clinical cohorts in order to identify specific proteins and novel proteoforms relevant to lipid metabolism, type 2 diabetes (T2D), and cardiovascular diseases (CVD). Using statistical methods, several clinically relevant correlations were identified, such as greater apolipoprotein A-I oxidation in patients with T2D and CVD compared to participants with diabetes but without CVD, or controls without diabetes [90]. Additionally, a unique truncation proteoform of SAA missing one *N*-terminal arginine (R) residue (des-R SAA) was lower in T2D [104].

Most significant were the findings related to apolipoproteins (apo) C-I, C-II and C-III analysis. ApoC-I, C-II and C-III are major apolipoproteins within triglyceride (TG)-rich particles that regulate multiple functions, and are linked to many lipid disorders [119]. ApoCs are known to exist in several proteoforms *in vivo* [120], but their influence on triglycerides and lipid metabolism are not well known. Quantitative MSIA was recently developed that enabled for simultaneous analysis of apoC-I, C-II and C-III, utilizing chicken egg lysozyme (Lys) as an IRS for quantification [107]. This single assay enabled for detection and analysis of total of 13 apoCs proteoforms (Supplementary Figure S1, Supplementary Table S2). The multiplexed MSIA for apoCs was applied in analysis of 204 samples from obese adolescent individuals. The results indicated that specific proteoforms of apoC-III, are associated with fasting plasma TG [91]. In the same samples, other proteoforms, or total apoC measures, did not present with such relationships. Measurement of apoC-III proteoforms can, therefore, be used to provide important insights into the biology of TG metabolism and in conditions such as obesity and metabolic syndrome.

These data are powerful examples of how MSIA can be applied for clinical assessment of protein biomarkers. Measuring proteoforms of candidate biomarkers and looking at the complete protein profile as a complement to the quantitative measure, can yield valuable new insight into disease risk assessment and therapeutic management.

## 5. Conclusions

Mass spectrometry-based assays have come a long way in the past few decades, and slowly but surely are finding their place in clinical laboratories. Efforts are being made in both bottom-up and top-down MS in order enable a productive biomarker profiling. Reliable protein profiling is of particular importance in the area of biomarker research where not only proteins, but different proteoforms need to be consistently identified and quantified across large patient cohorts. Advantages of current MS-based methodologies include improvements on the technological side in terms of low acquisition and operating costs, ease of use, ruggedness, and high throughput ability. When coupled with innovative sample preparation strategies and applied to important clinical problems, they have the potential to deliver rapid, sensitive, and cost-effective assays [121].

MSIA and similar affinity-based MS methodologies are paving their way into the clinical world. With proper clinical designs in place, MSIA undoubtedly has the potential to be emerged for analysis of more and more candidate protein biomarkers. The simple and streamlined workflow gives MSIA and similar MS-based methodologies a great advantage for clinical biomarker assaying. These assays provide the solid foundation for the future, which will aim to implement a novel perspective of the protein biomarker world, introducing simplicity for complex protein profiling.

## Figures and Tables

**Table 1:** Mass spectrometric immunoassays for analysis of human proteins exhibiting various proteoforms. The listing order is: single protein targets, multiplexed assays (with simultaneous analysis of two or more proteins) and multiple target analyses (population proteomics studies of multiple protein biomarkers).

Protein Target(s) *	MSIA Approach	Study	Reference
**Single protein assays**			
Apolipoprotein A-I	Qualitative	Clinical application	[ 90]
Apolipoprotein C-III	Quantitative	Clinical application	[ 91]
Beta 2-microglobulin	Qualitative Quantitative	Method development and application Method development	[ 92] [85]
Brain natriuretic peptide	Quantitative	Method development and application	[ 93]
C-peptide	Qualitative	Method development and application	[ 94]
C-reactive protein	Quantitative	Method development and application	[ 95]
Cystatin C	Qualitative Quantitative	Population proteomics Method development and application	[ 96] [97]
Vitamin D-binding protein	Qualitative	Clinical proteomics	[ 54,55,98]
Haptoglobin	Qualitative	Method development and application	[ 99]
Insulin-like growth factor 1	Quantitative	Method development Population proteomics	[ 88] [52]
Insulin	Qualitative Quantitative	Method development and application	[ 100]
Macrophage migration inhibitory factor	Quantitative	Method development	[ 50]
Osteocalcin	Qualitative	Method development and application	[ 53]
Parathyroid hormone-related protein	Qualitative	Method development and application	[ 101]
Regulated on activation, normal T cell expressed and secreted	Qualitative Quantitative	Clinical proteomics Method development	[ 102] [51]
Retinol-binding protein	Qualitative Quantitative	Method development and application Method development	[ 103] [86]
Serum amyloid A	Qualitative Quantitative	Method development and application Clinical application	[ 83] [104]
Serum amyloid P	Qualitative	Method development	[ 105]
Transthyretin	Quantitative	Method development	[ 106]
Multiplexed assays **			
Apolipoprotein C-I Apolipoprotein C-II Apolipoprotein C-III	Qualitative Quantitative	Method development Method development	[ 107]
Apolipoprotein A-I, Apolipoprotein A-II, Apolipoprotein E	Qualitative	Method development and application	[ 108]
Insulin-like growth factor 1, Insulin-like growth factor 2	Qualitative	Method development	[ 88,109]
Serum amyloid A, Transthyretin, Myoglobin	Qualitative	Method development and application	[ 110]
Multiple protein targets ***			
Transthyretin, Transferrin	Qualitative	Method application, population proteomics	[ 111]
Transthyretin, Retinol-binding protein	Qualitative	Method development and application	[ 87]
Multiple targets	Quantitative	Population proteomics	[ 112,113,114,115,116,117]
Multiple targets	Qualitative	Clinical proteomics	[ 89]

* Note that references in the table are presented alphabetically. The protein targets are labeled using full names; ** Multiplexed assays provide simultaneous analysis of several proteins in single run; *** Multiple protein target assays are applied in screening proteoforms from several proteins in separate assays.

## Supplementary Materials

The following are available online at www.mdpi.com/2227-7382/4/1/13/s1, Table S1: Serum Amyloid A (SAA) proteoforms identified using mass spectrometric immunoassay; Table S2: Apolipoproteins C-I (apoC-I), C-II (apoC-II) and C-III (apoC-III) proteoforms identified using mass spectrometric immunoassay; Figure S1. Liner mass spectra obtained apolipoproteins C-I, C-II and C-III proteoforms in human plasma sample using MSIA with checken-egg lysozyme as an IRS.

## Abbreviations

apoA-I	Apolipoprotein A-I
apoA-II	Apolipoprotein A-II
apoE	Apolipoprotein E
apoC-I	Apolipoprotein C-I
apoC-II	Apolipoprotein C-II
apoC-III	Apolipoprotein C-III
B2m	Beta 2-microglobulin
BNP	Brain natriuretic peptide
C-peptide	C-peptide
CRP	C-reactive protein
cysC	Cystatin C
GcG	Vitamin D-binding protein
IGF1	Insulin-like growth factor 1
IGF2	Insulin-like growth factor 2
MIF	Macrophage migration inhibitory factor
MYO	Myoglobin
PTH	Parathyroid hormone-related protein
RANTES	Regulated on Activation, Normal T Cell Expressed and Secreted
RBP	Retinol-binding protein
SAA	Serum amyloid A
TRFE	Transferrin
TTR	Transthyretin
