# Supplementary material for: Mass Spectrometric Immunoassays in Characterization of Clinically Significant Proteoforms
Source: Proteomes. 2016 Mar 17;4(1):13. doi: 10.3390/proteomes4010013 (PMC5217360; doi:10.3390/proteomes4010013)
Supplement: Supplementary file 1 [file proteomes-04-00013-s001.pdf]

Supplementary Materials: Mass Spectrometric Immunoassays in Characterization of Clinically Significant Proteoforms

Olgica Trenchevska, Randall W. Nelson and Dobrin Nedelkov

Table S1. Serum Amyloid A (SAA) proteoforms identified using mass spectrometric immunoassay.

SAA Proteoform		Theoretical MW	Observed MW
**Native SAA proteoforms** *			
SAA 1.1	native SAA proteoform 1.1	11,682.679	11,683.687
SAA 1.3	native SAA proteoform 1.3	11,654.626	11,655.186
SAA 2.1	native SAA proteoform 2.1	11,628.679	11,629.131
SAA 2.2	native SAA proteoform 2.2	11,647.725	11,648.639
**Truncated SAA proteoforms** **			
SAA 1.1 R	1.1 lacking 1 *N*-terminal (R-) amino acid	11,526.492	11,526.841
SAA 1.1 RS	1.1 lacking 2 *N*-terminal amino acids (RS-)	11,439.414	11,439.398
SAA 1.1 RSFF	1.1 lacking 4 *N*-terminal amino acids (RSFF-)	11,147.090	11,146.402
SAA 1.1 RSFFS	1.1 lacking 5 *N*-terminal amino acids (RSFFS-)	11,058.633	11,059.899
SAA 1.3 R	1.3 lacking 1 terminal (R-) amino acid	11,498.438	11,498.535
SAA 1.3 RS	1.3 lacking 2 terminal amino acids (RS-)	11,411.360	11,411.169
SAA 1.3 RSFF	1.3 lacking 4 *N*-terminal amino acids (RSFF-)	11,117.009	11,116.928
SAA 1.3 RSFFS	1.3 lacking 5 *N*-terminal amino acids (RSFFS-)	11,029.930	11,030.054
SAA 2.1 R	2.1 lacking 1 *N*-terminal (R-) amino acid	11,472.492	11,472.643
SAA 2.1 RS	2.1 lacking 2 *N*-terminal amino acids (RS-)	11,385.414	11,384.985
SAA 2.1 RSFF	2.1 lacking 4 *N*-terminal amino acids (RSFF-)	11,091.062	11,092.791
SAA 2.2 R	2.2 lacking 1 terminal (R-) amino acid	11,491.538	11,492.033
SAA 2.2 RS	2.2 lacking 2 terminal amino acids (RS-)	11,404.460	11,403.224

* Native SAA proteoforms represent the full-length SAA proteins derived from *SAA1* (SAA1.1 and SAA1.3) and *SAA2* (SAA2.1 and SAA2.2) genes; ** Truncated SAA proteoforms originate from the native SAA proteins, but are lacking one, or more amino acids from the *N*-terminus in the amino acid sequence.

Table S2. Apolipoproteins C-I (apoC-I), C-II (apoC-II) and C-III (apoC-III) proteoforms identified using mass spectrometric immunoassay.

ApoC Proteoform		Theoretical MW	Observed MW
apoC-I des-TP	apoC-I lacking TP residues	6432.344	6432.104
apoC-I native	apoC-I native	6630.565	6630.572
apoC-II des-TQQPQQ	apoC-II lacking terminal hexapeptide	8204.162	8205.290
apoC-III_{0a}	apoC-III native	8764.652	8764.587
apoC-II native	apoC-II native	8914.906	8915.760
apoC-III_0	apoC-III + $(Gal)_1(GalNAc)_1$	9135.800	9137.925
apoC-III_1 des-A *	apoC-III + $(Gal)_1(GalNAc)_1(NauAc)_1$ des-Ala	9350.171	9351.781
apoC-III_1	apoC-III + $(Gal)_1(GalNAc)_1(NauAc)_1$	9422.249	9422.562
apoC-III_2 des-A *	apoC-III + $(Gal)_1(GalNAc)_1(NauAc)_2$ des-Ala	9641.429	9642.267
apoC-III_2	apoC-III + $(Gal)_1(GalNAc)_1(NauAc)_2$	9712.507	9713.492
apoC-III var 4 **	apoC-III + $(Gal)_2(GalNAc)_2(Fuc)_3$	9933.768	9933.941
apoC-III var 5 **	apoC-III + $(Gal)_3(GalNAc)_3(Fuc)_2$	10,152.961	10,153.559
apoC-III var 6 **	apoC-III + $(Gal)_4(GalNAc)_2(Fuc)_3$	10,258.056	10,259.700
apoC-III var 7 **	apoC-III + $(Gal)_2(GalNAc)_4(Fuc)_3$	10,340.158	10,341.098
apoC-III var 8 **	apoC-III + $(Gal)_3(GalNAc)_3(Fuc)_4$	10,445.869	10,446.206
apoC-III var 9 **	apoC-III + $(Gal)_5(GalNAc)_3(Fuc)_4$	10,769.541	10,770.404

* Des-A apolipoprotein C-III proteoforms correspond to the truncated apoC-III proteoforms lacking one *N*-temrinal Alanine (des-A) residue in the amino acid sequence; ** Apolipoprotein C-III proteoforms labeled var 4 to var 9 represent glycosylated apoC-III derivatives, which contain Fucose (Fuc) in the glycan motif. Fucosylated apoC-III have recently been identified with detailed structural studies, and can also be identified using MSIA.

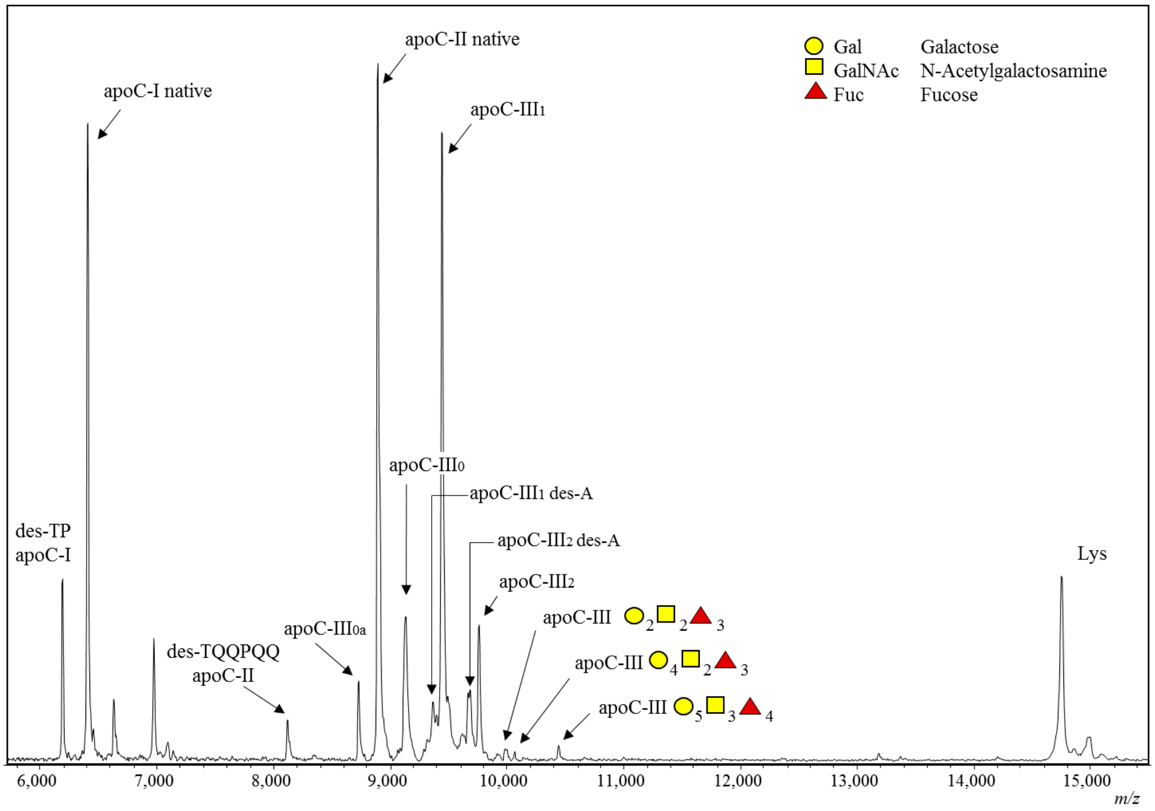

Figure S1. Liner mass spectra obtained apolipoproteins C-I, C-II and C-III proteoforms in human plasma sample using MSIA with chicken-egg lysozyme as an IRS.
